# Habitat Characteristics Predicting Distribution and Abundance Patterns of Scallops in D’Entrecasteaux Channel, Tasmania

**DOI:** 10.1371/journal.pone.0085895

**Published:** 2014-01-13

**Authors:** Tania Mendo, Jeremy M. Lyle, Natalie A. Moltschaniwskyj, Sean R. Tracey, Jayson M. Semmens

**Affiliations:** 1 Institute for Marine and Antarctic Studies, University of Tasmania, Hobart, Tasmania, Australia; 2 School of Environmental and Life Sciences, University of Newcastle, Ourimbah, New South Wales, Australia; The Australian National University, Australia

## Abstract

Habitat characteristics greatly influence the patterns of distribution and abundance in scallops, providing structure for the settlement of spat and influencing predation risk and rates of survival. Establishing scallop-habitat relationships is relevant to understanding the ecological processes that regulate scallop populations and to managing critical habitats. This information is particularly relevant for the D’Entrecasteaux Channel, south-eastern Tasmania (147.335 W, 43.220 S), a region that has supported significant but highly variable scallop production over many years, including protracted periods of stock collapse. Three species of scallops are present in the region; the commercial scallop *Pecten fumatus,* the queen scallop *Equichlamys bifrons*, and the doughboy scallop *Mimachlamys asperrima*. We used dive surveys and Generalized Additive Modelling to examine the relationship between the distribution and abundance patterns of each species and associated habitat characteristics. The aggregated distribution of each species could be predicted as a function of sediment type and species-specific habitat structural components. While *P. fumatus* was strongly associated with finer sediments and *E. bifrons* with coarse grain sediments, *M. asperrima* had a less selective association, possibly related to its ability to attach on a wide range of substrates. Other habitat characteristics explaining *P. fumatus* abundance were depth, *Asterias amurensis* abundance, shell and macroalgae cover. *Equichlamys bifrons* was strongly associated with macroalgae and seagrass cover, whereas *M. asperrima* abundance was greatly explained by sponge cover. The models define a set of relationships from which plausible hypotheses can be developed. We propose that these relationships are mediated by predation pressure as well as the specific behavioural characteristics of each species. The findings also highlight the specific habitat characteristics that are relevant for spatial management and habitat restoration plans.

## Introduction

The distribution and abundance of scallops are influenced by habitat characteristics such as depth, substrate type, currents, turbidity, and salinity (see review by [1]). At a finer spatial scale structural components of habitat, such as presence of polychaete tubes [2], hydroids [3]), sponges [4], macroalgae [5] and or shells [6], provide settlement substrates for settled scallop larvae or ‘spat’. Attachment by spat on structures can reduce predation rates [7], enhance growth - as an elevated position in the water column provides access to better quality food [8], and avoids smothering by soft sediments [9].

The value of habitat structure in reducing risk of predation continues into the juvenile and adult phase. Habitat characteristics greatly influence predation by affecting predation efficiency and predator-prey encounter rates [10], [11]. Predator encounters are reduced for juvenile bay scallops *Argopecten irradians* by attaching to the upper canopy of the eelgrass *Zostera marina* [7]. Complex habitats with greater numbers of horse mussels, sponges and ascidians provide refuge for *Pecten novaezelandiae* from predation by sea stars and gastropods [12]. Beyond directly reducing scallop visibility to predators, structure may impact movement and foraging behaviours of predators, as is the case with the queen scallop *Equichlamys bifrons* which suffer less predation mortality in seagrass beds than on bare sand because starfish have reduced mobility within the seagrass [13].

Despite the apparent importance of specific habitat characteristics in influencing scallop distribution and abundance patterns, quantitative studies on scallop-habitat relationships are rare. Identifying the habitat characteristics to which scallops are associated is relevant in managing, conserving, and even restoring these habitats. This type of information is particularly necessary for the D’Entrecasteaux Channel, south-eastern Tasmania, where three species of scallops co-occur; the commercial scallop *Pecten fumatus,* queen scallop *Equichlamys bifrons*, and doughboy scallop *Mimachlamys asperrima*. The D’Entrecasteaux Channel supported a significant commercial dredge fishery for scallops from the early 1920s to late 1960s, with catches peaking 4500 tonnes of meat in the mid 1960s and declining rapidly thereafter [14]. Significant depletions of scallop populations have occurred throughout the history of the fishery, resulting in area closures to allow for stock recovery. In 1990 the D’Entrecasteaux Channel was declared a recreational-only scallop fishery [15] but the fishery was closed shortly afterwards due to the lack of scallops. By the mid-2000s there was evidence of stock rebuilding, following more than a decade of fishery closure, that led to the area being reopened as a dive-only fishery in 2005, with a reduced daily bag limit of 40 scallops per person. Despite this ostensibly ‘conservative’ approach to management, the abundance of commercial scallops declined by approximately 80% between 2006 and 2010, due in part to the effects of fishing coupled with natural mortality and poor recruitment during this period [16].

The three co-occurring scallop species exhibited distinct and temporally consistent distribution patterns within the area during the 2000s [16], suggesting that species-specific habitat requirements may have an influence on their distribution. The abundance of scallops, however, has varied significantly from year to year, with variable and episodic recruitment experienced by each of the species. *Pecten fumatus* is found mainly on a range of soft sediment substrates including silt-sand and coarse sand [17], [18]. *Pecten fumatus* spat bysally attach to filamentous substrate such as macroalgae until approximately 6–10 mm in shell length when they release the byssus and then recess in the substrate [19]. *Equichlamys bifrons*, do not recess [20] and are often found in association with the seagrass *Heterozostera tasmanica* [13], [18]. *Mimachlamys asperrima,* bysally attach throughout their lifetime to a wide range of substrates such as bryozoans, seaweeds, sponges, oysters, mussels, old scallop shells, timber and rock [21].

An understanding of the relationships between habitat characteristics and the distribution and abundance patterns of each of these three species of scallops will provide insight into the ecological processes that regulate these populations. Being a relatively shallow and sheltered system, the D’Entrecasteaux Channel provided a unique opportunity to study the patterns of distribution by direct observation. In this study we have used dive surveys to examine the relationship between the distribution and abundance patterns of each species and associated habitat characteristics, including structural components, sediment type, predator abundance and depth. We hypothesize that specific habitat features influence the patterns of distribution of each species in different ways and discuss how these relationships are possibly mediated by predation pressure and the behavioural characteristics of each species.

## Materials and Methods

### Study Area

**Figure 1:**
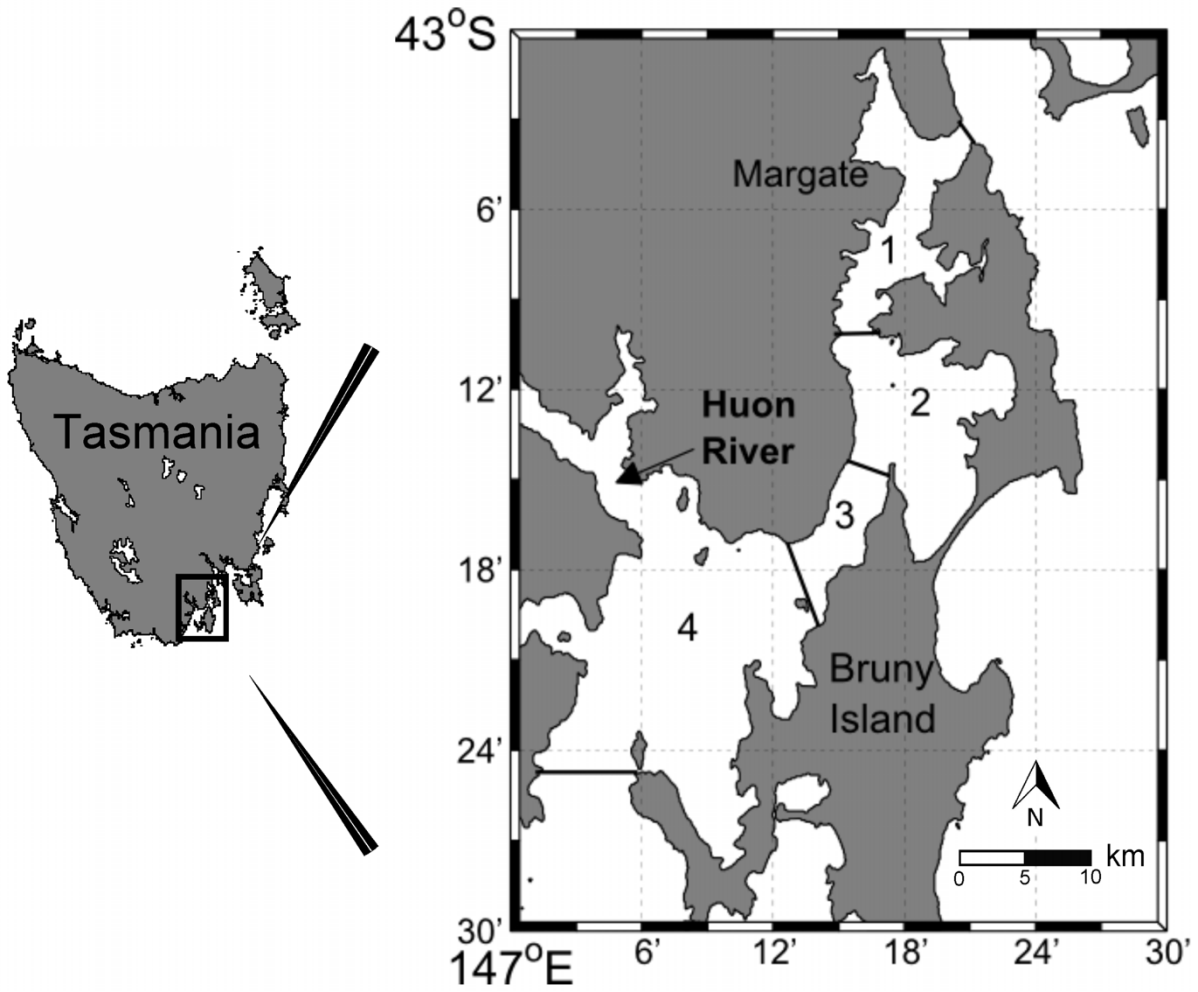
Map of the D’Entrecasteaux Channel. Numbers represent the Areas referred to throughout the manuscript.

The D’Entrecasteaux Channel (147.33590 W and 43.22028 S), separates Bruny Island from the Tasmanian mainland. It was divided into four sections based on topography and bathymetry: a narrow northern section with an average depth of 20 m (Area 1 in Fig. 1), an extensive shallow mid-section with an average depth of 15 m (Area 2), a narrow central area with stronger currents than the other Areas and an average depth of 14 m (Area 3) and a southern region with an average depth of 40 m which opens to the Southern Ocean (Area 4) [18], [22]. D’Entrecasteaux Channel (Channel) system is micro-tidal, with a spring tide ranging up to 1 m [22]. The study was conducted under the Authority of the Department of Primary Industries, Parks, Water and Environment (DPIPWE) permit No. 10028.

### Distribution Patterns

Scallop distribution and abundance in the Channel were quantified using dive surveys of 59 sites defined in [16]. The survey sites were restricted to depths <20 m and to soft sediments. Briefly, at each site, a 100 m transect was laid in a haphazard direction from the boat and two divers then searched and collected all scallops 1 m either side of the transect line covering an area of 200 m^2^. The species and shell length (largest distance parallel to the hinge) was recorded for each scallop collected. However, given the potential for very small scallops to be underrepresented due to collection bias based on size, analyses have been limited to include only individuals >30 mm. The numbers of two potential scallop predators, the native eleven-arm sea star *Coscinasterias muricata* and the invasive northern Pacific sea star *Asterias amurensis,* was also recorded for each transect.

Patterns of scallop abundance were analyzed by comparing them to a Poisson (random) distribution, which assumes that the expected number of organisms is the same in all sampling areas and is equal to the mean [23]. Agreement between observed and expected values was evaluated using a chi-square test of goodness of fit at the 5% level of significance, the null hypothesis being that the distribution did not differ significantly from a Poisson distribution [24]. To evaluate if the distribution was aggregated, the standardised Morisita’s Index of dispersion (I) was used because it is independent of population density and sample size [25]. This index ranges from −1 to +1, with zero indicating a random distribution pattern and negative values indicating a uniform distribution and positive values an aggregated distribution pattern [26]. Values >−0.5 and <0.5 are significant at the 5% level.

### Habitat Structural Components, Sediment Type and Depth

The main habitat structural components of the surveyed sites were macroalgae species (including seagrass), sponges, and shell debris. To generate semi-quantitative estimates of coverage, these structural components were ranked using a three-point scale of relative abundance. Sponges were ranked as being absent when none were recorded within the transect area, low when 1–10 sponges were counted and medium when more than 10 were present. Macroalgae and shell cover were estimated visually and when the component was not observed within the transect area it was ranked as absent, low when the coverage was judged to be less than about 10% and medium when the coverage was >10%. None of these components, however, had coverage levels in excess of 50%.

A sediment core, taken to a depth of approximately 2 cm, was collected by divers at each site for grain size assessment. Samples were dispersed using calgon (0.5% [mass:volume] sodium hexametaphosphate) [27] and then oven dried (60°C, 48 hours), weighed and shaken through a series of eight sieves ranging from 63 µm to 8 mm. The sediment in each sieved fraction was weighed to the nearest 0.1 gram and the cumulative percentage by weight of the eight fractions was calculated and the mean plotted against a phi (Ф) scale where:
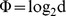
where *d* is particle diameter in millimeters. Mean grain size (*d_m_*) was estimated using phi values corresponding to the 16^th^, 50^th^ and 84^th^ percentiles of the cumulative proportion of weight using the formula:



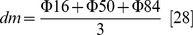
where larger *d_m_* values correspond to finer grain sizes [29]. Mean grain size was classified according to the Wentworth scale [29] which combines numerical intervals of grain size with rational definitions (pebble, sand, mud, etc) [30]. Water depth was measured at each site using dive computers within 0.1 m precision.

### Relationship between Abundance Patterns and Explanatory Variables

To visualize the spatial distribution of scallops, sea stars, mean grain size and depth, a triangle-based cubic interpolation algorithm was applied to fit an interpolated surface to the average value recorded for each site using Matlab [31]. Coastlines for maps were extracted from [32].

Scatterplots indicated than none of the continuous explanatory variables (mean grain size, depth, *A. amurensis* and *C. muricata* counts) were correlated. Scallop abundance was modelled as a function of explanatory variables using Generalized Additive Models (GAMs) [33]. Generalized Additive Models provide a flexible framework to model the relationship between abundance and environmental variables and have been applied to several marine organisms [34]–[36]. Generalized Additive Models were fitted using the mgcv package from the statistic software R [37], [38]. Explanatory variables were selected if significant (p<0.05). As the data were overdispersed a quasi-Poisson distribution was used [39]. Due to the tendency of GAMs to overfit the basis dimension parameter k was set to a maximum of 8 to correct for over fitting without compromising the model [38]. Categorical variables were analysed as ordered variables using orthogonal polynomial contrasts to examine trends and determine whether response variables changed linearly or nonlinearly as a function of habitat structural component cover [40].

Model selection was based on Generalized Cross Validation (GCV) [38], percentage deviance explained and visual examination of residuals. Spatial autocorrelation in the models’ residuals was investigated through Variogram analysis using the geoR package v 1.6-22 in R [41]. One of the model assumptions is there is no spatial autocorrelation. Violation of this assumption was tested by comparing a variogram of the deviance residuals with Monte Carlo envelope empirical variograms computed from 300 independent random permutations of the residuals [42]. There was no evidence of significant spatial autocorrelation on the residuals of any model as the semi-variance was within the boundaries of the Monte Carlo envelopes in the variograms.

## Results

### Distribution Patterns

**Figure 2:**
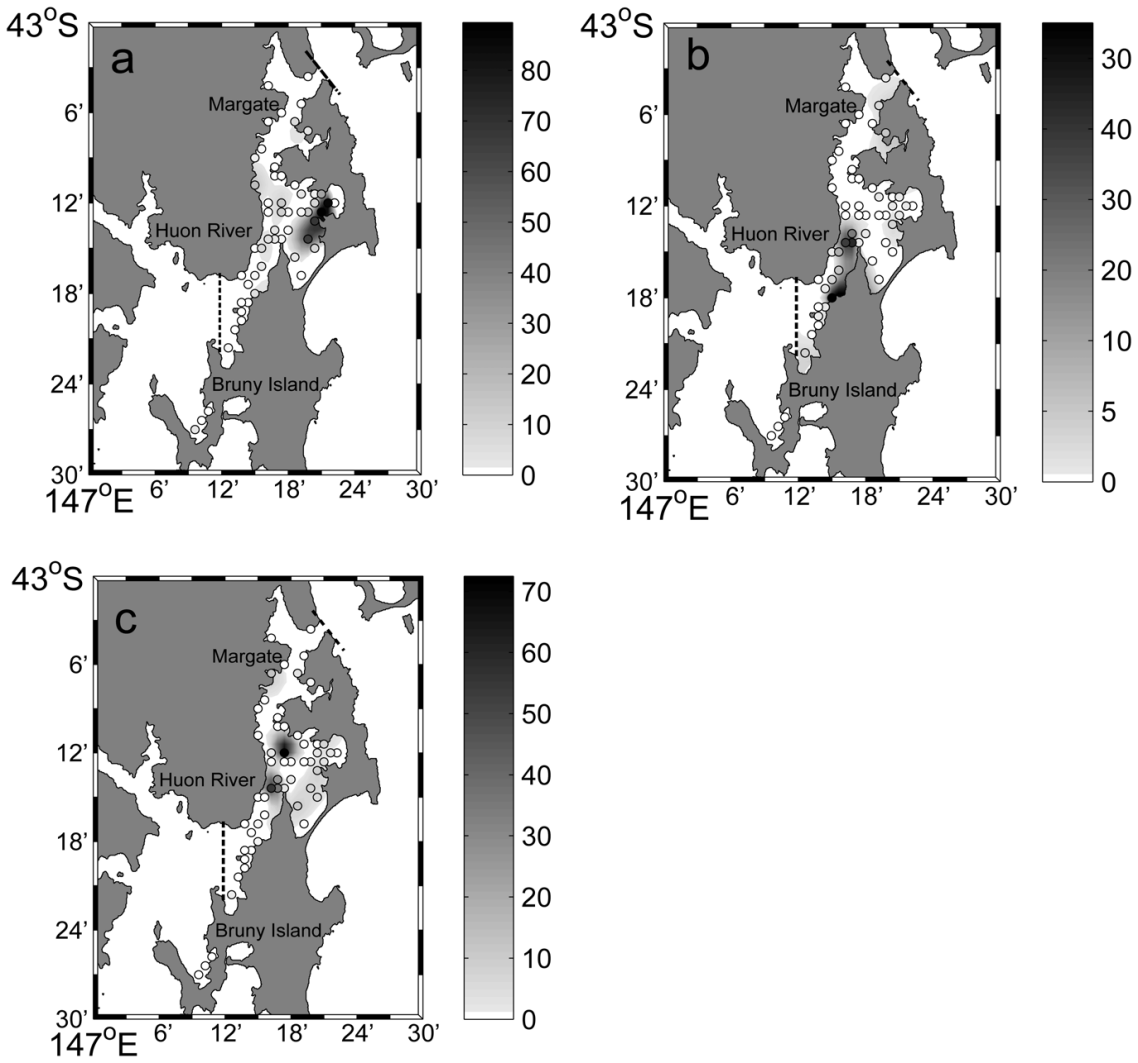
The interpolated distribution and densities of scallops per 100 m^2^. a) *Pecten. fumatus*, b) *Equichlamys bifrons* and c) *Mimachlamys asperrima* throughout the D’Entrecasteaux Channel in 2010. Circles indicate the survey sites and the colour intensity (white = no scallops) indicates the interpolated relative density of scallops. Note density scales (to the right of each map) differ among species. Areas located left of the dotted line were considered outside the model interpolation domain.

*Pecten fumatus* were most dense in the eastern section of Area 2, with a maximum of 85 scallops per 100 m^2^ but were very scarce in Areas 1, 3 and 4 (Fig. 2). *Equichlamys bifrons* were most dense in Area 3 with as many as 33 scallops per 100 m^2^, scarce in Areas 1, 2 and were absent in Area 4. *Mimachlamys asperrima* were found in the highest densities in Areas 2 and 3, with a maximum of 73 scallops per 100 m^2^, but were absent in Area 4. All three species had aggregated, non random distribution according to the Standarised Morisita’s Index (Table 1).

**Table 1:** Goodness of fit tests for a random (Poisson) or aggregated distribution (Morisita’s Standarised Index of Dispersion).

Test	*Pecten fumatus*	df	*Equichlamys bifrons*	df	*Mimachlamys asperrima*	df
Random distribution – Poisson	2976.5*	18	1350.6*	13	2131.3*	16
Test of aggregation – Morisita	0.555∧	58	0.546∧	58	0.558∧	58

* denotes significant difference from a Poisson distribution (p<0.05).

∧ denotes a significant departure from randomness at p<0.05.

**Figure 3:**
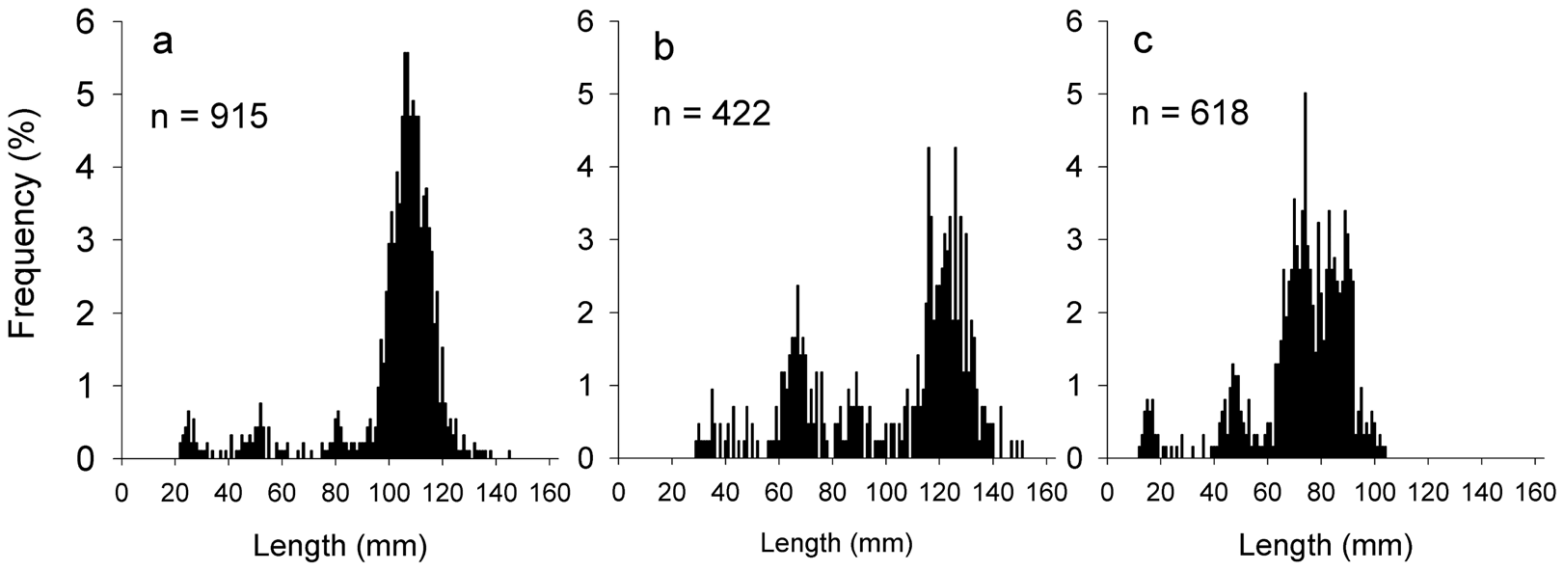
The size frequency (percent) distribution of scallops. (a) *Pecten fumatus*, (b) *Equichlamys bifrons,* and (c) *Mimachlamys asperrimus*. Samples were obtained from 59 sites within the D’Entrecasteaux Channel in 2010.

The size frequencies of the three species of scallops consisted of multimodal distributions and were dominated by large (adult) scallops (Fig. 3).

### Habitat Elements

**Figure 4:**
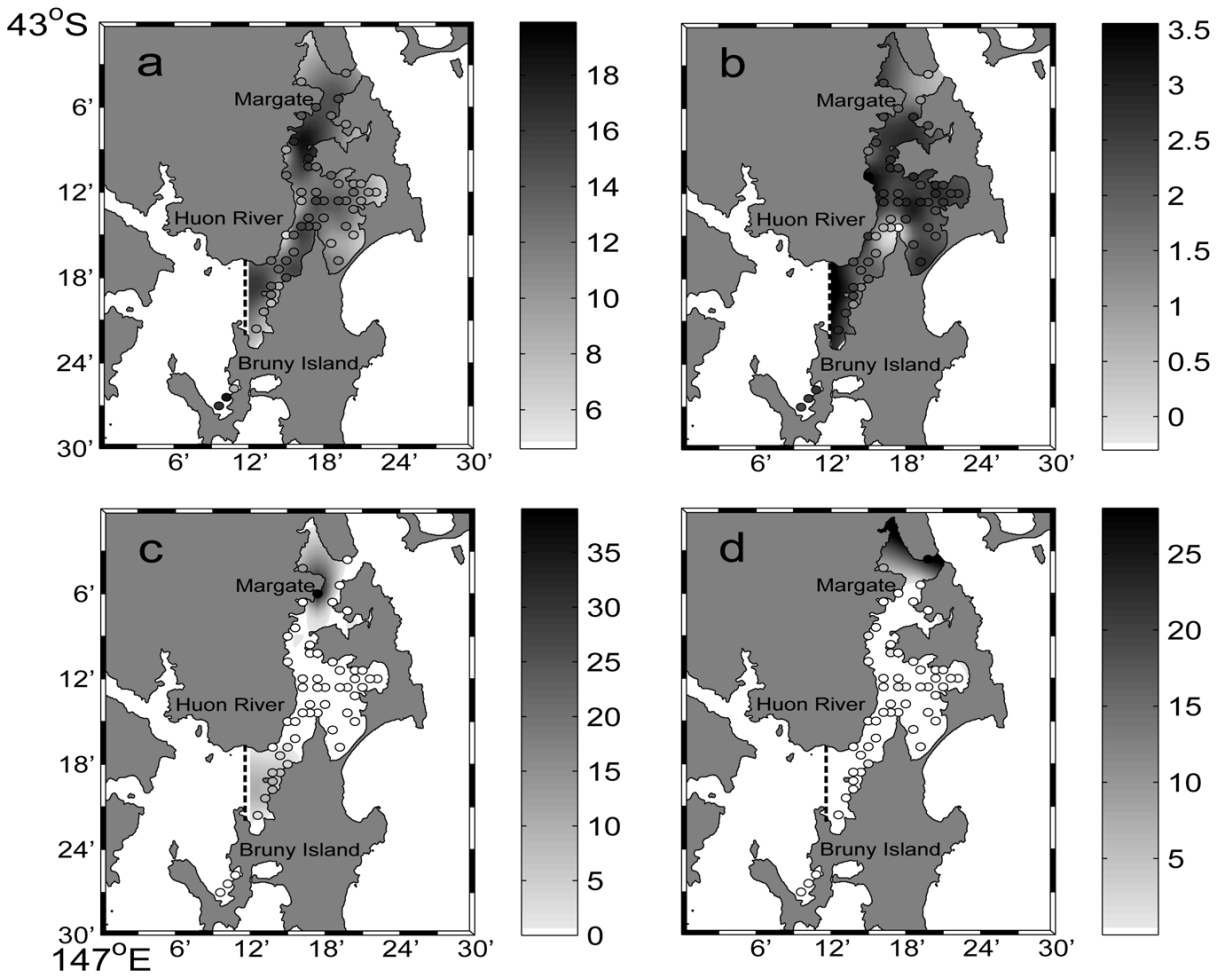
The interpolated values of a) depth in meters, b) mean grain size (mm) and abundances (number per 200 m^2^ transect) of c) *Asterias amurensis* and d) *Coscinasterias muricata* throughout the D’Entrecasteaux Channel in 2010. Circles indicate the survey sites and the colour intensity indicates the interpolated relative value. Note density scales vary between the starfish species. Areas located left of the dotted line were considered outside the model interpolation domain.

The 59 sites ranged from 5.6–18.9 m in depth. The deepest survey sites were located in Area 1, with an average depth of 13.2 meters, while the Area 2 sites were shallowest, averaging 9 m depth (Fig. 4a). Sites located in Areas 1, 2 and 4 were characterized by fine to very fine sand, while the northern section of Area 3 had coarse sand (Fig. 4b). The invasive northern Pacific sea star *Asterias amurensis* was found in 12 sites, mainly in the north west of Area 1 and in the south of Area 3 with 1–39 stars per transect. *Coscinasterias muricata* was only found in the northern end of the Channel, in six out of the 59 sites surveyed and usually in very low numbers (one site with 28 individuals per transect and the other five with a single individual per transect) (Fig. 4c and d, respectively).

**Figure 5:**
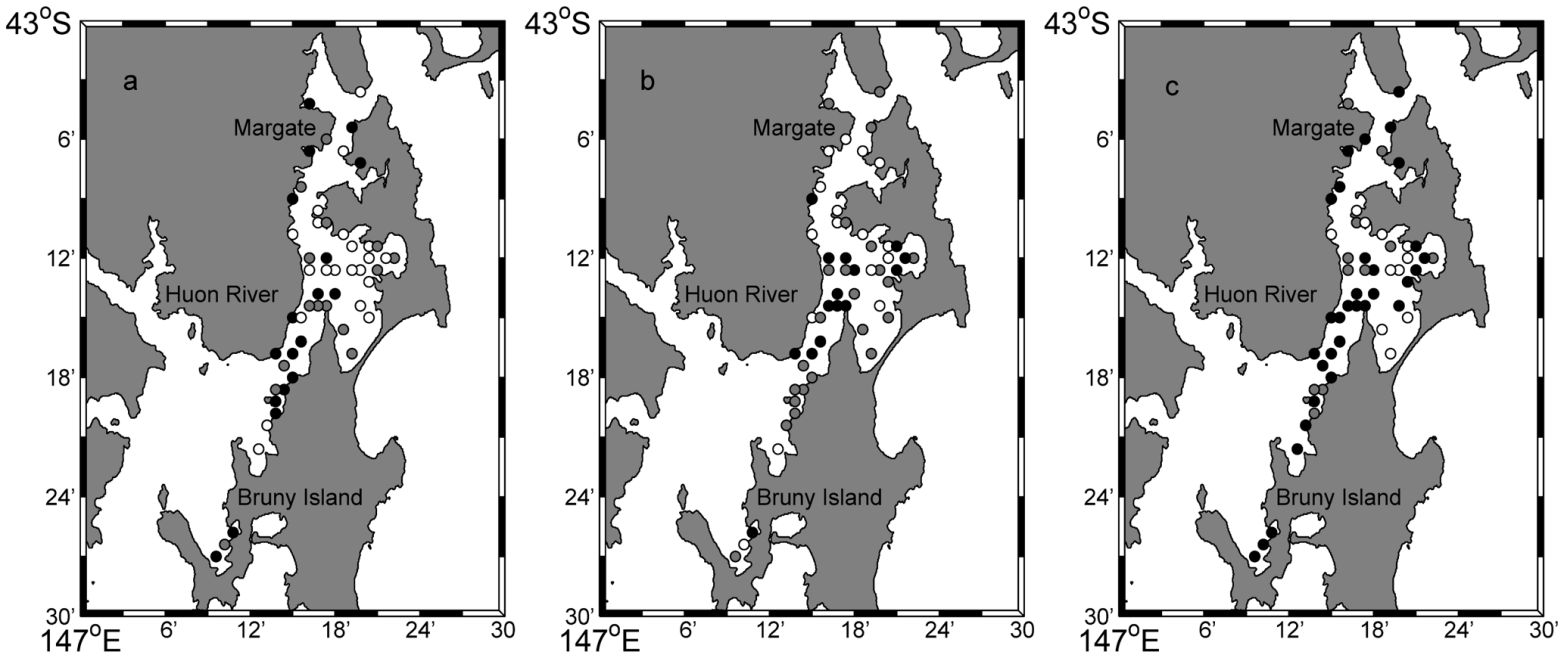
Distribution of main structural components in the D’Entrecasteaux Channel. a) sponges; b) shells and c) algae. Circle colours indicate percent cover, with absent (white), low (gray) and medium (black) cover.

Area 3 was characterized by a greater cover of habitat structural components (Fig. 5). Area 2 had less algae/seagrass cover than Areas 1, 3 and 4. Area 3 had more sites showing medium sponge cover than the other 3 Areas. There was no clear pattern in the distribution of shell cover (Fig. 5).

### Relationship between Scallop Abundance Patterns and Explanatory Variables

#### Commercial scallop *Pecten fumatus*

**Figure 6:**
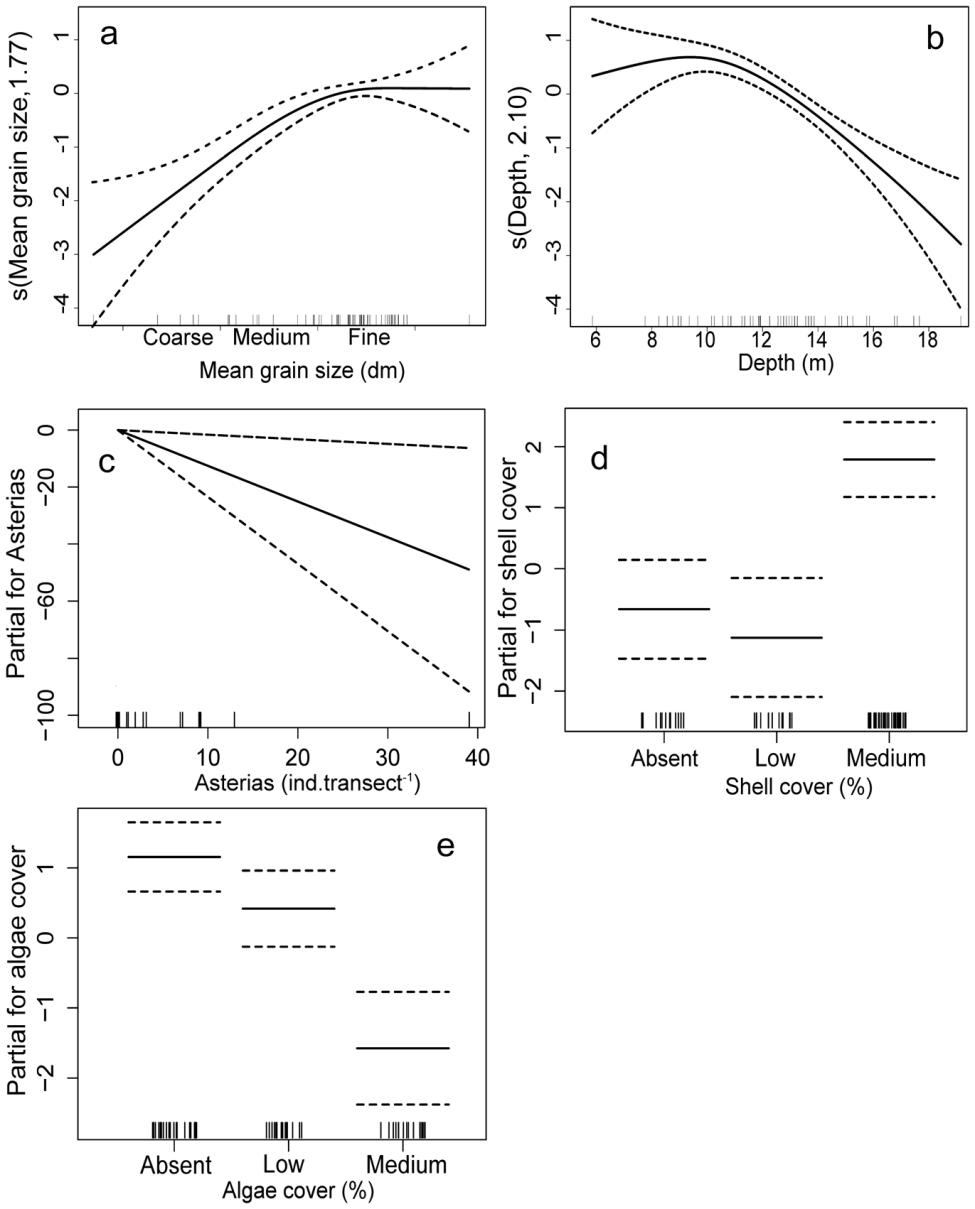
Graphical results of the GAM model fitted to *Pecten fumatus* abundance. Only significant variables are shown: a) mean grain size, b) depth, c) *Asterias amurensis* abundance, d) shell and e) algae/seagrass cover. The y-axis shows the relationship between the variable and scallop abundance, with effective degrees in freedom shown in brackets. Dashed lines represent 95% confidence intervals and whiskers on the x-axis indicate data presence.

Sediment size, depth, *A. amurensis* abundance, shell and macroalgae cover explained 72% of the difference in the abundance of *P. fumatus*. Greatest numbers of the species occurred in areas of fine sand and in depths from 8–12 meters (Fig. 6) and numbers increased with shell cover (significant 1^st^ and 2^nd^-order orthogonal polynomial contrast, t = 4.65, df = 1, p<0.001, and t = 2.31, df = 1, p = 0.024, respectively). In contrast, *P. fumatus* abundance decreased as macroalgal cover (significant 1^st^-order contrast t = −2.41, df = 1, p<0.001) and abundance of *A. amurensis* increased (t = −2.29, df = 57, p = 0.026).

#### Queen scallops *Equichlamys bifrons*

**Figure 7:**
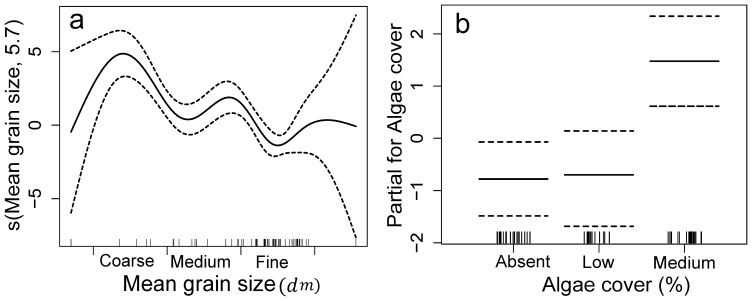
Graphical results of the GAM model fitted to *Equichlamys bifrons* abundance. Significant explanatory variables are a) mean grain size and b) algae/seagrass cover. See Fig. 6 for explanation.

Mean grain size and algae and seagrass cover explained 68.3% of the variation in *E. bifrons* abundance. The greatest numbers of were present in sites with medium to coarse sand (Fig. 7) and greater algae cover (significant 1^st^-order polynomial contrast, t = 3.37, df = 1, p = 0.001). There was no evidence that depth, shell, sponges and sea star abundance contributed to explaining variation in the abundance patterns of *E. bifrons*.

#### Doughboy scallop *Mimachlamys asperrima*

**Figure 8:**
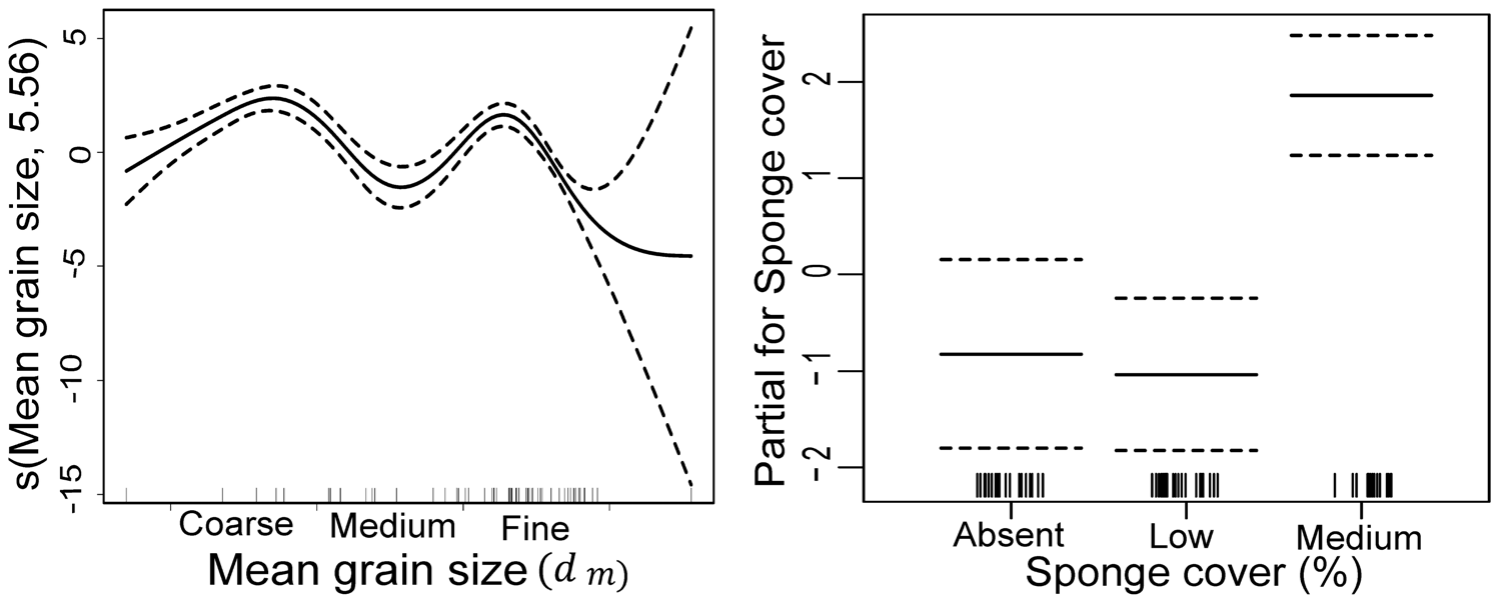
Graphical results of the GAM model fitted to *Mimachlamys asperrima* abundance. Significant explanatory variables are a) mean grain size and b) sponge cover. See Fig. 6 for explanation.

Mean grain size and sponge cover explained 69.7% of the variation in *M. asperrima* abundance (Fig. 8). Greater numbers were present in fine or coarse sand than in medium sand and the number of *M. asperrima* was highest with medium sponge cover (significant 1^st^ and 2^nd^-order orthogonal polynomial contrast, t = 3.73, df = 1, p<0.001, and t = 2.63, df = 1, p = 0.01, respectively). Depth, macroalgae, shells and sea star abundance did not contribute to explaining the patterns of variation in *M. asperrima* distribution.

## Discussion

Within the study area, each of the three scallop species exhibited aggregated rather than random or uniform patterns of distribution; aggregated distributions being typical amongst scallop species [43], [44]. While the spatial distribution patterns for each species were explained by sediment type, habitat structural components and/or presence of predators, the nature of the relationships between these factors and the distribution patterns differed markedly among species.

Across all species sediment type significantly explained scallop abundance. *Pecten fumatus* was more strongly associated with finer sediments, *E. bifrons* with coarse grain sediments, whereas *M. asperrima* had a less selective association with sediment type, possibly because this species is able to use byssal attachment on a wide range of substrates [45]. Habitat preferences are assumed to be adaptive, which means that associations between species and their habitats reflect enhanced survival and reproductive success in these particular habitats [46]. Differential abundance of bivalves based on sediment characteristics suggests differing refuge properties related to physical properties of the sediment or changes in predator-prey relationships [47], [48].

Prevalence of *P. fumatus* in fine sediments suggests that abundances may depend, in part upon increased survival in those sediments. The semi-burying or recessing behaviour of the juveniles and adults, in which the upper valve is level with or just below the surface of the sediment [1] is favoured in finer sediments compared to coarser sediments and provides protection from visual and non-visual predators, reduces fouling on the shell, and can anchor the individual in areas of strong currents [1]. Moreover, this behaviour does not interfere with active predator escape responses such as swimming [49].

While *P. fumatus* distribution was negatively associated with macroalgal/seagrass cover, *E. bifrons* had a positive relationship with macroalgae/seagrass cover that may be related to its use of this structural component as a refuge from predation. Predation rates in *E. bifrons* by sea stars have been shown to be lower in seagrass beds compared to bare sand and this is linked to the reduced mobility of sea stars within seagrass compared with over bare sand [13]. The positive relationship between *M. asperrima* and sponge abundances may be linked to the epizoic association between *M. asperrima* and sponges, including the red sponge (Crellidae family), the yellowish sponge (Myxillidae family), and the purple honeycomb sponge (*Equinochlathria* sp.) [50]. This association has the benefit that adhesion of the sea star *Coscinasterias muricata* tube feet on the scallop shell is reduced on sponges, effectively protecting the scallops from predation [50], [51]. To some extent the relationships between scallop abundance patterns and specific habitat characteristics can, therefore, be explained in terms of the benefits that these relationships afford in reducing predation pressure for each species.

The abundance of *Coscinateris muricata* did not explain distribution and abundance patterns of scallops. On the other hand, greater abundances of *P. fumatus* occurred where the invasive sea star *Asterias amurensis* was in relatively low numbers or absent. This sea star was first recorded in Tasmanian waters in 1986 [52] and its expansion within the Channel raised concerns about their potential impact on the endemic scallop populations. Outbreaks of this species had detrimental impacts on the shellfish industry in Japan [53] and losses of *P. fumatus* spat over a settlement season due to *A. amurensis* predation may be as much as 50% in Tasmania (S. Crawford pers. comm. in [54]). The negative relationship between the invasive *A. amurensis* and the scallop *P. fumatus*, but not the other two species of scallops may be due to habitat-mediated changes in predation risk [55]. Vulnerability to predation can vary in a species-specific manner within habitat types even among species that are morphologically and phylogenetically similar [56]. For instance, the probabilities of encountering scallops and predation success rates for a related predator, *Asterias vulgaris*, were influenced by particle size [57]. In the present study, the nature of the relationship between *A. amurensis* and *P. fumatus* abundance is unclear and we cannot rule out preferential habitat use by *A. amurensis* or interactions with other sources of prey, such as the distribution of other epi-benthic bivalves [58], as explanatory factors for the sea star abundance.

This study has demonstrated that macroalgae and seagrass, shell and sponge cover have important roles in determining adult scallop distributions. However, it is uncertain when these distribution patterns are established, whether at settlement and/or as a result of post-settlement processes. Scallop spat have distinct habitat requirements due to their need to attach to structural elements. Therefore, the habitat characteristics associated with settlement might be very different to those observed for the adults as observed by Howarth et al [59] for *Pecten maximus* and *Aequipecten opercularis.* Information is needed about habitat specificity during the attached and unattached stage concurrently to identify if habitat associations vary ontogenetically and therefore, if different habitats need to be included in management plans.

This study provides clear descriptions of the relationships between habitat characteristics and species-specific patterns of abundance in three scallop species; with sediment type and habitat structural components being of major importance. These associations do not imply direct causal or functional relationships, however, and the mechanisms or processes behind these associations are not clear. Spatial variation in distribution patterns of adults may result from a number of factors such as among-habitat variation in larval arrival and settlement [60], [61], differential availability of shelter from predation (i.e. habitat complexity) [62], or agonistic interactions with conspecifics or competitors [63]. To understand the underlying mechanisms explaining distribution patterns the various components of recruitment need to be examined concurrently. Manipulative experiments in which predation rates are compared amongst habitats for the three species could help understand the relative importance of predation and behaviour traits in regulating population size in different habitats. From this a better understanding of the relative importance of settlement and post-settlement processes in regulating population size in different habitats will be possible [64].

The spatial distribution patterns for the three species of scallops were explained by sediment type, habitat structural components and/or presence of predators, however, the nature of the relationships between these factors and the distribution patterns differed markedly among species. Generating predictive relationships between species and habitat characteristics is important because they provide insight into ecological processes that regulate populations as well as defining those habitat characteristics that need to be considered in developing spatial management and/or restoration plans (i.e. fishing in a way that allows structure to re-establish).
